# Clinical, Metabolic, and Sonographic Predictors of Endometrial Carcinoma Among Women Presenting With Postmenopausal Bleeding: A Prospective Observational Study From a Tertiary Care Center in South India

**DOI:** 10.7759/cureus.93820

**Published:** 2025-10-04

**Authors:** Sunitha Vijayasingh, Nithya R, Reshmi S, Meena T S

**Affiliations:** 1 Obstetrics and Gynecology, Sree Balaji Medical College & Hospital, Chennai, IND

**Keywords:** clinical, endometrial ca, gynecological malignancy, histopathology (hp), postmenopausal woman

## Abstract

Background

Endometrial carcinoma represents the most common gynecological malignancy among postmenopausal women, with rising incidence attributed to increasing rates of obesity, metabolic syndrome, and changing reproductive patterns. Identifying clinical and metabolic risk factors is essential for early diagnosis and improved outcomes. This study aims to evaluate the clinical, demographic, and metabolic risk factors associated with endometrial carcinoma in postmenopausal women presenting with vaginal bleeding and to correlate bleeding patterns, endometrial thickness (ET), and histopathological findings.

Methods

This prospective observational study was conducted over 18 months at a tertiary care teaching hospital in South India. Ninety-one postmenopausal women with vaginal bleeding underwent detailed clinical evaluation, transvaginal sonography for ET assessment, and endometrial sampling for histopathological diagnosis. Demographic data, reproductive history, comorbidities, and bleeding patterns were systematically recorded. Statistical analysis was performed using the chi-square test, with a significance threshold of p < 0.05.

Results

Of the 91 women studied, 16 (17.6%) were diagnosed with endometrial malignancy, while 75 (82.4%) had benign pathology. Malignant cases were significantly associated with higher socioeconomic status, early menarche (10-12 years), late menopause (≥55 years), recurrent postmenopausal bleeding, and positive family history of malignancy. Obesity (BMI ≥30 kg/m²) and combined diabetes with hypertension showed a strong association with malignancy. All malignant cases exhibited ET >5 mm on sonography. Atypical hyperplasia and endometrioid adenocarcinoma were the predominant malignant histologies observed.

Conclusions

Obesity, metabolic syndrome, reproductive factors, and recurrent bleeding are major risk determinants for endometrial carcinoma in postmenopausal women. Integrating clinical, metabolic, and sonographic criteria can enhance early detection and risk stratification. Focused screening and prevention strategies targeting these risk factors are crucial for reducing disease burden and improving gynecologic care outcomes.

## Introduction

Endometrial carcinoma has emerged as the most prevalent gynecological malignancy in high-income countries and stands as the second most common in several developing nations, including India [1]. This increasing burden is largely attributed to ongoing lifestyle transitions, the rising prevalence of obesity, and delayed menopause in women [1]. Globally, the disease constitutes a significant health concern, accounting for approximately 417,000 new cases and over 97,000 deaths each year, with steadily rising trends over the past decade [2]. Unlike ovarian or cervical cancers, which often remain silent in early stages, endometrial carcinoma generally presents at an early stage due to its hallmark symptom: postmenopausal bleeding (PMB). PMB serves as a conspicuous clinical warning sign, demanding immediate medical evaluation since up to 10-20% of women presenting with PMB may be diagnosed with an underlying endometrial malignancy [3].

PMB is defined as any bleeding from the genital tract that occurs after 12 consecutive months of amenorrhea in a woman who has reached menopause. This clinical event is regarded as a sentinel symptom and a critical red flag, often pointing toward endometrial pathology ranging from benign conditions such as atrophy to premalignant lesions like hyperplasia and, most concerningly, carcinoma [4]. Among the various differential diagnoses, endometrial carcinoma holds the gravest implication and therefore warrants thorough and timely investigation. It is also essential to distinguish PMB from abnormal uterine bleeding (AUB), which occurs across all reproductive age groups and during perimenopause, since PMB has a much stronger correlation with malignancy and should not be conflated with bleeding disturbances seen during the menopausal transition.

The pattern of bleeding in postmenopausal women offers important diagnostic cues. Clinically, PMB may present as a single isolated episode, as intermittent recurrent spotting, or as continuous bleeding. Continuous or recurrent bleeding is often suggestive of an underlying structural abnormality or malignant disease, while a solitary episode is more likely to be attributed to benign causes, particularly endometrial atrophy. Nevertheless, even a single episode of bleeding in a postmenopausal woman cannot be dismissed without proper evaluation, as malignancy may still be detected [5]. Thus, analyzing bleeding patterns and stratifying patients accordingly can help clinicians prioritize diagnostic urgency and streamline patient management by directing high-risk individuals toward earlier interventions.

Several risk factors have been strongly associated with the development of endometrial carcinoma. These include conditions that promote prolonged unopposed estrogen exposure, such as obesity, diabetes mellitus, polycystic ovarian syndrome, late menopause, early menarche, nulliparity, and hereditary syndromes such as Lynch syndrome [6]. Among these, obesity has consistently been highlighted as one of the most significant contributors. This association is biologically explained by increased peripheral conversion of androgens to estrogens via aromatase activity in adipose tissue, particularly in postmenopausal women [7]. The global obesity epidemic, currently affecting more than 650 million individuals, has had a profound impact on the epidemiology of endometrial cancer. It is estimated that nearly 40% of all cases can be attributed directly to excess body weight [8]. Moreover, diabetes mellitus and metabolic syndrome act synergistically with obesity to magnify the risk through mechanisms including hyperinsulinemia, insulin resistance, chronic low-grade inflammation, and unregulated cellular proliferation [9].

In the Indian context, the burden of endometrial carcinoma presents unique challenges. Awareness regarding the significance of PMB as a red-flag symptom remains limited, and access to gynecologic services is often delayed. Consequently, many women present at advanced stages of the disease when treatment options are more invasive and the prognosis is less favorable. Unlike cervical cancer, for which established population-based screening programs exist, endometrial carcinoma lacks routine screening modalities, underscoring the critical role of symptom-based detection and early evaluation. Risk stratification, particularly in women presenting with PMB, becomes essential in such a resource-limited setting. Assessing clinical profile, comorbidities such as obesity and diabetes, bleeding characteristics, and imaging findings can optimize diagnostic precision and resource utilization.

Among diagnostic modalities, transvaginal sonography (TVS) has become a pivotal first-line investigation in women presenting with PMB. Endometrial thickness (ET) measurement through TVS is a widely accepted, noninvasive, and easily accessible tool. Numerous studies have validated an ET cutoff of 4 mm, below which the risk of malignancy is extremely low and beyond which endometrial biopsy or sampling is strongly recommended [10]. Despite its utility, ET measurement alone is insufficient for comprehensive risk stratification, particularly in women with metabolic risk factors or recurrent bleeding. In such cases, a multidimensional diagnostic approach integrating clinical, metabolic, sonographic, and histopathological factors may substantially improve early detection rates.

Given this backdrop, the present study was designed to evaluate the clinical and metabolic risk factors associated with endometrial carcinoma in women presenting with PMB at a tertiary care hospital in South India. Furthermore, the study sought to examine correlations among bleeding patterns, ET, and histopathological findings. By identifying high-risk subgroups and refining diagnostic algorithms, the study aims to contribute toward improving early detection strategies, facilitating timely treatment, and ultimately enhancing outcomes for women at risk of endometrial carcinoma.

## Materials and methods

Study design and setting

This prospective observational study was conducted in the Department of Obstetrics and Gynecology at Sree Balaji Medical College & Hospital (SBMCH), Chennai, a tertiary care teaching hospital in South India. The study was carried out over a period of 18 months, from September 2023 to February 2025, following approval from the Institutional Human Ethics Committee, SBMCH (002/SBMCH/IHEC/2023/2017).

Study population

The study included postmenopausal women who presented to the gynecology outpatient department with vaginal bleeding during the study period. PMB was defined as any uterine bleeding occurring after 12 consecutive months of amenorrhea in women aged 45 years or above who were not on hormone therapy. Only women who had attained natural menopause and subsequently experienced vaginal bleeding were considered for recruitment.

Inclusion criteria

Women were eligible if they had spontaneous cessation of menstruation for at least 12 months, presented with one or more episodes of PMB, and were willing to undergo complete clinical, radiological, and histopathological evaluation.

Exclusion criteria

Women were excluded if they had visible lesions on the vulva, vagina, or cervix on clinical examination; were on hormone replacement therapy or tamoxifen; had a history of endometrial carcinoma, pelvic radiation, or prior hysterectomy; or had bleeding due to coagulation disorders, anticoagulant therapy, or iatrogenic causes such as cervical or vaginal trauma. Premenopausal and perimenopausal women with AUB, as well as asymptomatic postmenopausal women with incidentally detected thickened endometrium on imaging or abnormal cytology on Pap smear, were also excluded. Women unwilling to provide informed consent or undergo investigations were not included.

Sample size and sampling

The required sample size was estimated using the formula for a single population proportion with precision. Based on an anticipated prevalence of 26% from a previous study [11], a precision of 10%, and a 95% CI, the minimum sample size was calculated to be 91 participants. A purposive sampling method was used, enrolling all eligible women who presented sequentially during the study period until the sample size requirement was met.

Data collection procedure

All participants underwent a detailed clinical evaluation. A structured and pretested proforma was used to document demographic characteristics such as age, socioeconomic background, using the Modified B. G. Prasad Scale [12], and educational status. Reproductive factors, including age at menarche, parity, and age at menopause, were recorded, along with medical comorbidities such as obesity, diabetes mellitus, hypertension, and hypothyroidism. Past medical history of breast cancer, tamoxifen or hormone replacement therapy use, and family history of gynecologic or colorectal malignancy were also noted. Special emphasis was placed on characterizing the bleeding pattern, which was classified into three groups: a single isolated episode, intermittent recurrent bleeding separated by periods of amenorrhea, or continuous bleeding lasting seven or more consecutive days after menopause.

All participants underwent TVS to assess ET, measured in millimeters at the thickest point of the double-layer endometrial echo. Women with an ET less than or equal to 5 mm were considered low risk and managed conservatively, though they were followed up with repeat imaging if bleeding persisted. Women with an ET greater than 5 mm underwent endometrial sampling. The threshold of 5 mm for evaluation of PMB is widely accepted in international guidelines, including those from the American College of Obstetricians and Gynecologists (ACOG), which recommend endometrial sampling in women with PMB when ET is >5 mm on TVS [11]. This cutoff is also consistent with institutional practice and has been validated in multiple studies as a sensitive marker for detecting endometrial carcinoma. Pipelle biopsy was the first-line method used for endometrial tissue acquisition. In cases where pipelle sampling was technically unsuccessful or yielded insufficient tissue, dilatation and curettage or hysteroscopy-guided biopsy under anesthesia was performed. Hysteroscopy was repeated in women with recurrent bleeding despite previously benign histological findings.

The histopathological evaluation of tissue specimens was carried out by senior pathologists who were blinded to clinical and imaging details. Histological diagnoses were grouped as benign, which included atrophic endometrium, polyps, inflammatory lesions, and hyperplasia with or without atypia, or malignant, which included endometrioid adenocarcinoma, serous carcinoma, clear cell carcinoma, and carcinosarcoma. Anthropometric measurements were recorded, and body mass index was calculated and classified according to World Health Organization criteria into overweight (25-29.9 kg/m²) and obese (≥30 kg/m²). Clinical details of diabetes mellitus, hypertension, and other chronic illnesses were verified from medical records and current medication use.

Statistical analysis

Data were entered in Microsoft Excel and analyzed using IBM SPSS Statistics for Windows, Version 25.0 (Released 2017; IBM Corp., Armonk, NY, USA). Quantitative variables such as age, body mass index, and ET were expressed as mean with SD, while qualitative variables, including bleeding patterns, comorbidities, and histopathological outcomes, were expressed as frequencies and percentages. Associations between categorical variables were assessed using the chi-square test or Fisher’s exact test as appropriate. A two-tailed p-value of less than 0.05 was considered statistically significant.

## Results

Among the 91 women studied, the majority were between 55 and 60 years of age, accounting for 26 (28.6%). Based on the Modified B. G. Prasad classification, most belonged to the lower socioeconomic class (26, 28.6%), followed by the lower middle class (22, 24.2%). Age at menarche was commonly between 10 and 12 years in 41 (45.1%) participants, while menopause most frequently occurred between 50 and 54 years in 35 (38.5%). The duration since menopause was 3-9 years in 39 (42.9%) women. A single episode of PMB was the most common presentation in 60 (65.9%). Diabetes mellitus with or without hypertension was observed in 39 (42.9%) women, whereas 22 (24.2%) had no systemic disorder. The predominant BMI category was 25-29.9 kg/m² in 28 (30.8%). A family history of malignancy was present in 26 (28.6%) (Table 1).

**Table 1:** Sociodemographic characteristics of the study population (n = 91) ^* ^Modified B. G. Prasad Scale ^#^ WHO Classification of BMI DM, diabetes mellitus; HTN, hypertension; PMB, postmenopausal bleeding

Characteristic	Parameter	n (%)
Age at presentation	≤45	4 (4.4)
	46-49	19 (20.9)
	50-54	25 (27.5)
	55-60	26 (28.6)
	>60	17 (18.7)
Socioeconomic status^*^	Upper	12 (13.2)
	Upper middle	13 (14.3)
	Middle	18 (19.8)
	Lower middle	22 (24.2)
	Lower	26 (28.6)
Age at menarche	<10 years	1 (1.1)
	10-12 years	41 (45.1)
	13-15 years	40 (44.0)
	>15 years	9 (9.9)
Age at menopause	<40 years	1 (1.1)
	40-44 years	3 (3.3)
	45-49 years	24 (26.4)
	50-54 years	35 (38.5)
	55-60 years	26 (28.6)
	>60 years	2 (2.2)
Duration since menopause	<3 years	32 (35.2)
	3-9 years	39 (42.9)
	≥10 years	20 (22.0)
Pattern of PMB	Single episode	60 (65.9)
	Recurrent	31 (34.1)
Systemic comorbidities	Diabetes mellitus	20 (22.0)
	Hypertension	17 (18.7)
	DM + HTN	19 (20.9)
	Hypothyroidism	13 (14.3)
	No disorder	22 (24.2)
BMI (kg/m²)^#^	<18.5	25 (27.5)
	18.5-24.9	26 (28.6)
	25-29.9	28 (30.8)
	30-34.9	6 (6.6)
	35-39.9	4 (4.4)
	≥40	2 (2.2)
Family history of malignancy	Yes	26 (28.6)
	No	65 (71.4)

On sonographic evaluation, the majority of women had an ET >5 mm, seen in 85 (93.4%), while only 6 (6.6%) had a thickness ≤5 mm. Histopathological examination revealed that the most common finding was atrophic endometrium in 24 (26.4%), followed by disordered proliferative endometrium in 19 (20.9%), proliferative phase in 9 (9.9%), polyp in 16 (17.6%), and hyperplasia without atypia in 12 (13.2%). Among premalignant and malignant conditions, atypical hyperplasia was observed in 10 (11.0%) cases and endometrial carcinoma in one (1.1%) case (Table 2).

**Table 2:** Sonographic and histopathological findings among the study population (n = 91) ET, endometrial thickness

Finding	Parameter	N (%)
Sonographic findings	ET ≤5 mm	6 (6.6%)
	ET >5 mm	85 (93.4%)
	Total	91 (100%)
Histopathological findings	Proliferative phase	9 (9.9%)
	Disordered proliferative	19 (20.9%)
	Polyp	16 (17.6%)
	Hyperplasia without atypia	12 (13.2%)
	Atrophic	24 (26.4%)
	Atypical hyperplasia	10 (11.0%)
	Endometrial carcinoma	1 (1.1%)
	Total	91 (100%)

**Figure 1:**
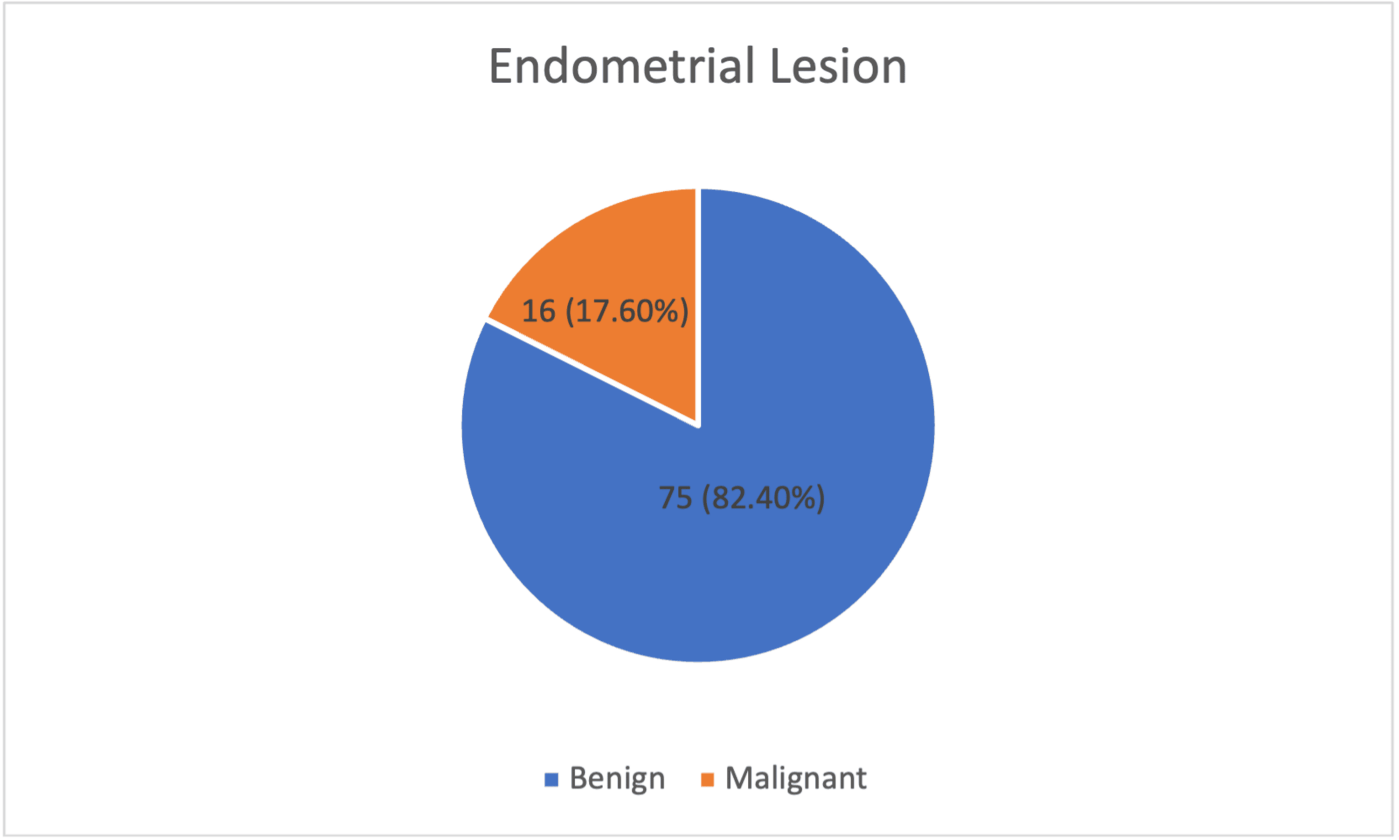
Distribution of benign and malignant endometrial lesions on histopathology (n = 91) Data are represented as n (%).

Out of 91 women evaluated, histopathology revealed benign endometrial lesions in 75 (82.4%) cases. Malignant changes were identified in 16 (17.6%) cases. Thus, the majority had benign pathology, with a smaller but significant proportion showing malignancy (Figure 1).

Out of 91 women evaluated, several variables showed significant associations between benign and malignant endometrial pathology. Malignancy was more frequent in women from higher socioeconomic classes, particularly Class I, with seven cases (43.8%) compared to only five benign cases (6.7%). Age at menarche of 10-12 years showed higher malignant cases with 11 (68.8%) versus 30 benign (40.0%). Similarly, late menopause between 55 and 60 years was significantly associated with malignancy, with nine cases (56.3%) compared to 17 benign (22.7%). Recurrent PMB was strongly linked to malignancy, present in 11 women (68.8%) versus 20 benign (26.7%). Among comorbidities, combined diabetes and hypertension was associated with malignant pathology in 10 women (62.5%) compared to nine benign (12.0%). Higher BMI categories were also significant, with no benign cases but six malignant (37.5%) in BMI 30-34.9, four malignant (25.0%) in BMI 35-39.9, and two malignant (12.5%) in BMI ≥40. A positive family history of malignancy was observed in nine malignant cases (56.3%) compared to 17 benign cases (22.7%). On ultrasound, all malignant cases (100%) had ET >5 mm. Histopathology revealed atypical hyperplasia in 10 malignant cases (62.5%), with none in benign cases, while atrophic endometrium was more common in benign cases, with 24 (32.0%) (Table 3).

**Table 3:** Association of clinical, demographic, and histopathological variables with benign and malignant endometrial findings (n = 91) ^*^ Modified B. G. Prasad Scale ^# ^WHO Classification of BMI ^@^ Chi-square/Fisher’s exact test A p-value < 0.05 is statistically significant. DM, diabetes mellitus; ET, endometrial thickness; HTN, hypertension; PMB, postmenopausal bleeding

Variable/category	Benign, n (%)	Malignant, n (%)	Chi-square value^@^	p-Value
Age at presentation				
≤45 years	4 (5.3%)	0 (0%)	0.311	0.577
46-49 years	18 (24.0%)	1 (6.3%)	1.737	0.188
50-54 years	20 (26.7%)	5 (31.3%)	0.366	0.545
55-60 years	20 (26.7%)	6 (37.5%)	0.862	0.353
>60 years	13 (17.3%)	4 (25.0%)	0.480	0.489
Socioeconomic status^*^				
Class I	5 (6.7%)	7 (43.8%)	4.176	0.041
Class II	10 (13.3%)	3 (18.8%)	2.809	0.060
Class III	15 (20.0%)	3 (18.8%)	2.625	0.072
Class IV	20 (26.7%)	2 (12.5%)	1.975	0.160
Class V	25 (33.3%)	1 (6.3%)	1.502	0.240
Age at menarche				
<10 years	1 (1.3%)	0 (0%)	0.050	0.824
10-12 years	30 (40.0%)	11 (68.8%)	4.098	0.043
13-15 years	35 (46.7%)	5 (31.3%)	0.675	0.411
>15 years	9 (12.0%)	0 (0%)	1.606	0.205
Age at menopause				
<40 years	1 (1.3%)	0 (0%)	0.050	0.824
40-44 years	3 (2.7%)	0 (0%)	0.174	0.677
45-49 years	21 (28.0%)	3 (18.8%)	0.360	0.548
50-54 years	32 (42.7%)	3 (18.6%)	2.159	0.142
55-60 years	17 (22.7%)	9 (56.3%)	6.174	0.013
>60 years	1 (1.3%)	1 (6.3%)	1.126	0.289
Duration since menopause				
<3 years	31 (41.3%)	1 (6.3%)	2.595	0.107
3-9 years	30 (40.0%)	9 (56.3%)	1.497	0.221
≥10 years	14 (18.7%)	6 (37.5%)	2.763	0.096
Pattern of PMB				
Single episode	55 (73.3%)	5 (31.3%)	8.320	0.004
Recurrent	20 (26.7%)	11 (68.8%)		
Systemic comorbidities				
DM	18 (24.0%)	2 (12.5%)	0.680	0.41
HTN	15 (20.0%)	2 (12.5%)	0.415	0.520
DM + HTN	9 (12.0%)	10 (62.5%)	8.098	0.004
Hypothyroidism	12 (16.0%)	1 (6.3%)	0.800	0.370
No disorder	21 (28.0%)	1 (6.3%)	3.444	0.063
BMI (kg/m²)^#^				
<18.5	24 (32.0%)	1 (6.3%)	3.260	0.07
18.5-24.9	25 (33.3%)	1 (6.3%)	3.580	0.058
25-29.9	26 (34.7%)	2 (12.5%)	1.708	0.191
30-34.9	0 (0%)	6 (37.5%)	13.345	<0.001
35-39.9	0 (0%)	4 (25.0%)	9.497	0.002
≥40	0 (0%)	2 (12.5%)	4.827	0.028
Family history of malignancy				
Yes	17 (22.7%)	9 (56.3%)	6.173	0.013
No	58 (77.3%)	7 (43.7%)		
Ultrasound findings				
ET ≤5	6 (8.0)	0 (0)	5.18	0.023
ET >5	69 (92.0)	16 (100)		
Fractional curettage/Pipelle endometrial sampling				
Proliferative phase	9 (12)	0	12.4	0.205
Disordered proliferative endometrium	14 (18.67)	5 (31.25)	8.22	0.14
Polyp	16 (21.33)	0	6.14	0.07
Hyperplasia without atypia	12 (16)	0	23.1	0.205
Atrophic endometrium	24 (32)	0	54.1	0.004
Atypical hyperplasia	0	10 (62.50)	22.4	<0.001
Endometrial carcinoma	0	1	31.2	0.176

## Discussion

Our prospective observational study among 91 postmenopausal women with bleeding provides valuable insight into the multifactorial etiology of endometrial carcinoma. The most striking determinants identified were obesity, metabolic syndrome, early menarche, delayed menopause, family history of malignancy, and recurrent episodes of bleeding, all of which emerged as important risk factors for malignant endometrial pathology.

Obesity was the most powerful modifiable risk factor. The majority of malignant cases occurred among women with elevated BMI, and malignancy rates rose progressively with increasing BMI categories. This finding is consistent with Schmandt et al., who highlighted obesity as a central determinant of endometrial cancer, mediated both by adipose-driven estrogen production and by indirect pathways involving hyperinsulinemia and chronic inflammation [13]. Allen et al. in the EPIC cohort further demonstrated that each 5 kg/m² increase in BMI nearly doubled the risk of endometrial cancer [14]. Our findings corroborate these results, particularly given the absence of malignancy in women with BMI below the overweight range.

The biological plausibility of this association is reinforced by Zeleniuch-Jacquotte et al., who showed that elevated postmenopausal estrogen and androgen levels were predictive of endometrial cancer [15]. Similarly, Schmandt et al. confirmed obesity as a key determinant of postmenopausal endometrial cancer in their study of US radiologic technologists [13]. These global findings align with our observations, suggesting that adiposity-driven hormonal and metabolic disturbances play a central role in carcinogenesis.

Early menarche and late menopause also contributed significantly to malignancy risk, echoing the hormonal theory advanced by Thombre Kulkarni et al. and Linkov et al., who emphasized that prolonged lifetime estrogen exposure increases susceptibility [16,17]. Allen et al. additionally demonstrated, through EPIC data, that higher circulating estradiol is an independent predictor of endometrial cancer risk [14]. Our findings fit within this hormonal framework, highlighting the relevance of reproductive history in determining long-term risk.

Metabolic syndrome, particularly the coexistence of diabetes and hypertension, was strongly associated with malignancy in our cohort, even though individual conditions alone were not significant predictors. This observation aligns with Trabert et al., who reported that metabolic syndrome doubled the risk of endometrial carcinoma, particularly when multiple metabolic components coexisted [18].

Inflammatory pathways are also likely relevant. Dossus et al. demonstrated that obesity-related inflammatory markers such as CRP and IL-6 are linked to higher endometrial cancer risk [19]. While our study did not measure inflammatory markers, the predominance of malignancy in obese women with metabolic syndrome indirectly supports this pathway. Lifestyle-related factors may also contribute, as Friedenreich et al. found that physical activity reduces cancer risk, including endometrial carcinoma, by improving weight, metabolic health, and hormonal balance [20]. Although activity levels were not assessed in our series, the sedentary lifestyle observed in many malignant cases may have played a role, consistent with Bhaskaran et al., who linked high BMI to elevated risk across multiple cancer types, including endometrial carcinoma [21].

Histological subtypes may further influence risk associations. Yang et al. reported that metabolic factors are most strongly linked with endometrioid carcinomas (type I) [22], which were predominant in our series. This strengthens the biological coherence of our results. Mechanistic studies further point to hyperinsulinemia and altered adipokines as key drivers. Gunter et al. found that elevated insulin and IGF-I independently predicted endometrial cancer, while Cust et al. highlighted the protective role of adiponectin [23,24]. Though these markers were not measured in our population, the observed clustering of diabetes, hypertension, and obesity in malignant cases supports this metabolic-inflammation hypothesis.

Other clinical parameters also showed relevant patterns. Recurrent bleeding was significantly more frequent among malignant cases, underlining its importance as a clinical warning sign. Higher socioeconomic status was associated with greater malignancy risk, possibly reflecting lifestyle and dietary differences. Increasing age and longer duration since menopause showed trends toward higher malignancy rates but did not reach statistical significance in our sample.

Limitations of this study include its relatively small sample size and single-center design, which limit generalizability. The observational design precludes causal inferences, and detailed lifestyle factors such as physical activity and diet were not systematically assessed. Additionally, metabolic and inflammatory markers were not measured, restricting mechanistic exploration. Recall bias in self-reported reproductive history and comorbidities is possible, and the lack of long-term follow-up prevented outcome assessment. Larger multicenter studies with more diverse populations and comprehensive metabolic profiling are warranted to validate and expand these findings.

Implications of our study are clinically relevant. Recognition of obesity, metabolic syndrome, prolonged estrogen exposure, and recurrent bleeding as significant risk factors for endometrial carcinoma highlights the importance of early screening and preventive interventions in high-risk women. Lifestyle modification, weight reduction, and management of metabolic comorbidities could substantially reduce the endometrial cancer burden. At the same time, heightened clinical vigilance for recurrent PMB can aid in earlier detection, particularly in populations undergoing rapid lifestyle transitions.

## Conclusions

This study underscores the significant interplay between metabolic, reproductive, and clinical factors in determining endometrial cancer risk among postmenopausal women presenting with vaginal bleeding. Obesity, metabolic syndrome, early menarche, late menopause, family history of malignancy, and recurrent PMB emerged as key risk factors for malignant endometrial pathology in our cohort. The strong association of malignancy with higher BMI and clustered metabolic comorbidities highlights the urgent need for targeted risk reduction strategies, including lifestyle modification and early metabolic screening in at-risk populations. The findings also reinforce the importance of meticulous evaluation of bleeding patterns and individualized risk assessment, rather than reliance on a single clinical parameter. Although the study was limited by sample size and setting, its results are consistent with global evidence and provide a foundation for refining diagnostic pathways in resource-limited settings. Enhanced awareness, timely gynecological assessment, and integration of clinical, metabolic, and sonographic criteria can facilitate earlier detection and improve outcomes for women at risk of endometrial carcinoma. Future broader screening initiatives will be essential to further reduce the burden of this increasingly prevalent malignancy.
